# Response to COVID-19 mRNA vaccination in multiple myeloma is conserved but impaired compared to controls

**DOI:** 10.1186/s13045-021-01183-2

**Published:** 2021-10-13

**Authors:** Samuel Bitoun, Julien Henry, Christelle Vauloup-Fellous, Nicolas Dib, Rakiba Belkhir, Lina Mouna, Candie Joly, Delphine Desjardins, Marie Bitu, Roger Le Grand, Raphaèle Seror, Anne-Marie Roque Afonso, Xavier Mariette

**Affiliations:** 1 Rheumatology Department, Hôpital Bicêtre, Assistance Publique-Hôpitaux de Paris, FHU CARE, 78 Avenue du general Leclerc, 94270 Le Kremlin-Bicêtre, France; 2 Center for Immunology of Viral, Auto-immune, Hematological and Bacterial Diseases (IMVA-HB/IDMIT), Université Paris-Saclay, Inserm, CEA, Fontenay-aux-Roses, Le Kremlin-Bicêtre, France; 3 Virology Department, Université Paris-Saclay, INSERM U1193, AP-HP, Hôpital Paul Brousse, Villejuif, France

**Keywords:** Multiple myeloma, Daratumumab, SARS-COV-2, Vaccine, COVID-19, Neutralization

## Abstract

Patients with multiple myeloma are at high risk of severe forms of COVID-19. Despite data showing diminished response to vaccine, the era of highly efficient mRNA vaccine might be a gamechanger. We sought to examine response to mRNA vaccine between healthy controls (*n* = 28) and multiple myeloma (MM) patients (*n* = 27). Response was analyzed 1 month after the second dose of anti-SARS-CoV-2 BNT162b2 vaccine. Multiple myeloma patients showed diminished levels of Anti-Spike IgG levels compared to controls, but with a high proportion of patients achieving a humoral response (89% vs. 97% in controls). Neutralizing antibodies were present in 74% of patients versus 96% of controls. Patients under current daratumumab treatment had neutralizing activity of anti-SARS-CoV-2 antibodies. Multiple myeloma patients show diminished response to SARS-COV-2 vaccine but with still high response rate. The main potential risk factor of non-response to COVID-19 vaccine was uncontrolled disease under treatment.

**Supplementary Information:**

The online version contains supplementary material available at 10.1186/s13045-021-01183-2.

To the Editor,

We have read with great interest the article by Pimpinelli et al. [1], on immunogenicity of anti‑SARS‑CoV‑2 BNT162b2 vaccine in patients with myeloma malignancy. We performed a case control study to compare anti-spike IgG response and neutralizing activity of anti-SARS-CoV-2 antibodies in healthy controls (HC) and multiple myeloma (MM) patients  one month after the second  BNT162b2 dose. We analyzed factors associated with non-response in MM patients.

Patients and HC were vaccinated with BNT162b2 at days 0 and 28. Serological assessment (anti-Spike IgG) of vaccine response was performed at day 56 (Elecsys Anti-SARS-CoV-2 Cobas, Roche Diagnostics; cut off: 0.4 IU/mL), as well as neutralizing anti-Spike antibodies (iFlash-2019-nCoV Nab assay, Ylho; cut-off: 24 IU/ml). Patients with detectable levels of anti-nucleocapsid or positive SARS-COV-2 PCR at any time points were excluded. We have previously established a threshold ≥ 50 UI/ml of anti-Spike IgG which was related to neutralizing activity of anti-SARS-CoV-2 antibodies in 98% of subjects.

We recruited 37 consecutive patients with MM and 28 controls (8 males/20 females; median age: 58 years, range: 26–88 years) between January and March 2021. We excluded 10 patients: 4 contracted COVID-19 before the second dose and 1 one after, 4 were anti N+ at the second dose, 1 patient died. Among these excluded patients 3 patients died due to COVID-19 infection.

In the 27 remaining patients (Table 1), titers of anti-Spike IgG were significantly lower in MM patients than in HC (mean ± SD: 172.8 ± − 107.6 vs. 235 ± 57.6 *p* = 0.0013 Mann–Whitney test; Fig. 1a) at day 56. At the same timepoint, 24/27 (88.9%) patients with MM and 27/28 (96.4%) controls had detectable anti-Spike IgG (*p* = 0.35). Considering the threshold of 50 IU/ml of anti-Spike IgG to evaluate neutralizing activity of anti-SARS-CoV-2 antibodies, there was a trend towards a lower response rate in the MM group compared to HC (74.1% vs. 92.9%; *p* = 0.07, Fisher’s exact test). Neutralizing anti-SARS-CoV-2 antibodies were detected in 18/24 (75%) of MM patients and 25/26 (96.1%) of HC (*p* = 0.045 Fischer test Fig. 1b). We confirmed that no patient with a titer less than 50 IU/ml of anti-Spike IgG had neutralizing anti-Spike antibodies and found a robust correlation between anti-Spike IgG and neutralizing anti-Spike antibodies (*r* = 0.69 *p* ≤ 0.0001 Spearman test) (Additional file 1: Fig. S1).

**Table 1:** Comparison of positive neutralizing response and negative anti-SARS-CoV-2 spike protein antibody groups

	Responders: ≥ 50 IU/mL at day 56	Non responders: negative or < 50 IU/mL at day 56	*P* value
Number of patients	20	7	
Sex *n* (%)			0.09
Female (*n* = 15)	9 (60)	6 (40)	
Male (*n* = 12)	11 (91.7)	1 (8.3)	
Age in years, median (range)	69 (46–93)	74 (47–86)	0.65
Previous lines of therapy, median (range)	2 (1–8)	5 (2–11)	0.03
Previous autologous HSCT, *n* (%)			0.65
Had HSCT (*n* = 9)	6 (66.7)	3 (33.3)	
Did not have HSCT (*n* = 18)	12 (66.7)	6 (33.3)	
Therapy status *n* (%)			0.03
On therapy (*n* = 17)	10 (59)	7 (41)	
Not on therapy (*n* = 10)	10 (100)	0 (0)	
Residual polyclonal gammaglobulins (g/l), median (range)	4.9 (2–16.6)	2.4 (1.2–3.8)	0.0009
Disease status (per IMWG criteria) *n* (%)			
Myeloma in complete response or in very good partial response or partial response (*n* = 24)	20 (83.3)	4 (16.7)	0.002
Myeloma in progressive or stable disease under treatment (*n* = 3)	0	3 (100)	
Type of therapy, *n* (%)			
Daratumumab-based therapy (*n* = 9)	6 (67)	3 (33)	0.65
Non-Daratumumab-based therapy (*n* = 18)	14 (77.8)	4 (22.2)	

*HSCT* Hematopoietic stem-cell transplantation, *IMWG* International Myeloma Working Group

**Fig. 1:**
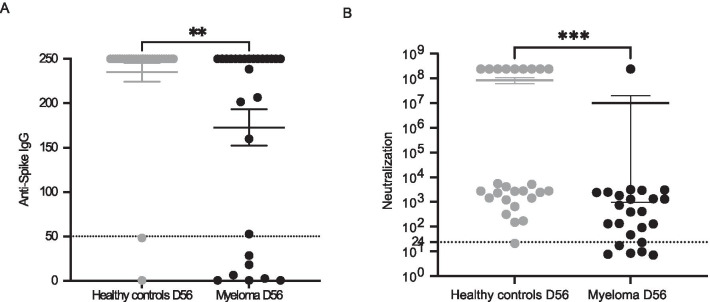
**a** Anti-spike IgG levels compared at day 56 after first vaccine injection between multiple myeloma patients and healthy controls. *p* = 0.0013 using Mann–Whitney test. **b** Neutralizing activity of anti-SARS-CoV-2 antibodies compared at day 56 after first vaccine injection between multiple myeloma patients and Healthy controls. *p* = 0.0002 using Mann–Whitney test

The main predictive factor of absence of response was MM disease status. Effectively, the only three MM patients with no detectable anti-spike IgG had a progressive or stable MM on therapy (none on daratumumab, Table 1). Progressive or stable disease was associated with a worse response to vaccine taking both detectable (3/3 vs. 0/24; *p* = 0.0003 Fischer’s exact test) or ≥ 50 IU/ml anti-spike IgG thresholds (3/7 vs. 20/20; *p* = 0.01; Fischer test). Residual gammaglobulin level was significantly higher in responders (≥ 50 IU/mL) compared to non-responders (6.1 ± 3.9 vs. 2.3 ± 0.8 mean ± SD *p* = 0.0009 Mann–Whitney test). Conversely, we found that daratumumab was not associated with worse immunogenicity, since 6/9 (66.7%) patients receiving daratumumab had anti-spike titers ≥ 50 UI/ml versus 14/18 (78%) not receiving daratumumab (*p* = 0.65).

Using a different assay to detect neutralizing anti-SARS-CoV-2 antibodies, we found a higher response rate in the MM group compared to Terpos et al. [2], but they assessed neutralization after only one injection. Compared to Pimpinelli et al. [1] who assessed immunogenicity after the second injection [1], we had similar response rates (74.1% vs. 78.6% respectively), even though we assessed response a little later (4 vs. 2 weeks after the second dose). However, contrarily to 2 other publications [1, 3] we did not confirm the negative impact of daratumumab on vaccine immunogenicity even though there was a numerical decrease of responders with neutralizing antibodies in those patients (66.7% vs. 78%). This might be due to lack of power in our study. It might also be due to the fact that most of our patients treated with daratumumab were in complete response and we have shown, as others, that active disease was a risk factor of low immunogenicity [3]. Thus, we also identified MM disease status (progressive or stable disease on treatment) regardless of treatment and low residual gammaglobulin level, as the main potential factors of non-response which is in line with was found by Terpos et al. [2] and Van Oekelen et al. [3]. The impact of MM status was not assessed by Pimpinelli et al.

Overall, these results support a diminished response to 2 doses of anti‑SARS‑CoV‑2 BNT162b2 vaccine in MM patients compared to HC. Similarly to the previous studies of the literature [1–3], a high proportion of MM patients still achieve a detectable humoral response: 89% in MM versus 97% in HC and neutralizing antibodies: 75% in MM vs 96% in HC. This is to be compared to no response at all in patients treated with anti-CD20-based regimen in chronic lymphocytic leukemia which received this drug in the previous year [4]. Even if most of MM patients achieved humoral immunization, diminished titers of anti-Spike and neutralizing antibodies are of a concern in the era of the delta variant. We identified uncontrolled MM on treatment as the main potential risk factor of non-response to COVID-19 vaccine. This requires confirmation in lager studies. In these patients without response, it is crucial to vaccinate their family members and primary caregivers. The use of a third dose should be evaluated in MM patients.

## Supplementary Information

**Additional file 1**.** Supplementary figure 1**. Correlation between anti-Spike IgG (Elecsys Anti-SARS-CoV-2 S, Cobas, Roche Diagnostics; cut off: 0.4 IU/ml) and neutralizing activity of anti-SARS-CoV-2 antibodies (iFlash-2019-nCoV Nab assay, Ylho; cut-off: 24 IU/ml) of Multiple myeloma and control patients. The threshold of 50 IU/mL with the anti-Spike IgG assay represents the limit established on a separate cohort, above which 98% of sample tested have detectable neutralizing activity of anti- SARS-CoV-2 antibodies as defined by the threshold of 24 IU/ml with the neutralization assay. r = 0.69 p ≤ 0,0001 Spearman test.

## Data Availability

The datasets used and/or analyzed during the current study are available from the corresponding author on reasonable request.

## Abbreviations

HC: Healthy controls
HSCT: Hematopoietic stem-cell transplantation
IMWG: International Myeloma Working Group
MM: Multiple myeloma
