# Supplementary material for: Response to COVID-19 mRNA vaccination in multiple myeloma is conserved but impaired compared to controls
Source: J Hematol Oncol. 2021 Oct 13;14:166. doi: 10.1186/s13045-021-01183-2 (PMC8512646; doi:10.1186/s13045-021-01183-2)
Supplement: Supplementary file 1 — Additional file 1. Supplementary figure 1. Correlation between anti-Spike IgG (Elecsys Anti-SARS-CoV-2 S, Cobas, Roche Diagnostics; cut off: 0.4 IU/ml) and neutralizing activity of anti-SARS-CoV-2 antibodies (iFlash-2019-nCoV Nab assay, Ylho; cut-off: 24 IU/ml) of Multiple myeloma and control patients. The threshold of 50 IU/mL with the anti-Spike IgG assay represents the limit established on a separate cohort, above which 98% of sample tested have detectable neutralizing activity of anti- SARS-CoV-2 antibodies as defined by the threshold of 24 IU/ml with the neutralization assay. r = 0.69 p ≤ 0,0001 Spearman test. [file 13045_2021_1183_MOESM1_ESM.docx]

Supplementary figure 1

Correlation between anti-Spike IgG (Elecsys Anti-SARS-CoV-2 S, Cobas, Roche Diagnostics; cut off: 0.4 IU/ml) and neutralizing activity of anti-SARS-CoV-2 antibodies (iFlash-2019-nCoV Nab assay, Ylho; cut-off: 24 IU/ml) of Multiple myeloma and control patients. The threshold of 50 IU/mL with the anti-Spike IgG assay represents the limit established on a separate cohort, above which 98% of sample tested have detectable neutralizing activity of anti-SARS-CoV-2 antibodies as defined by the threshold of 24 IU/ml with the neutralization assay. r=0.69 p=<0,0001 Spearman test.
